# Supplementary material for: Competition between microtubule-associated proteins directs motor transport
Source: Nat Commun. 2018 Apr 16;9:1487. doi: 10.1038/s41467-018-03909-2 (PMC5902456; doi:10.1038/s41467-018-03909-2)
Supplement: Supplementary file 1 — Supplementary Information [file 41467_2018_3909_MOESM1_ESM.pdf]

Competition between microtubule-associated proteins directs motor transport

Monroy *et al.*

Supplementary Figures and Figure Legends

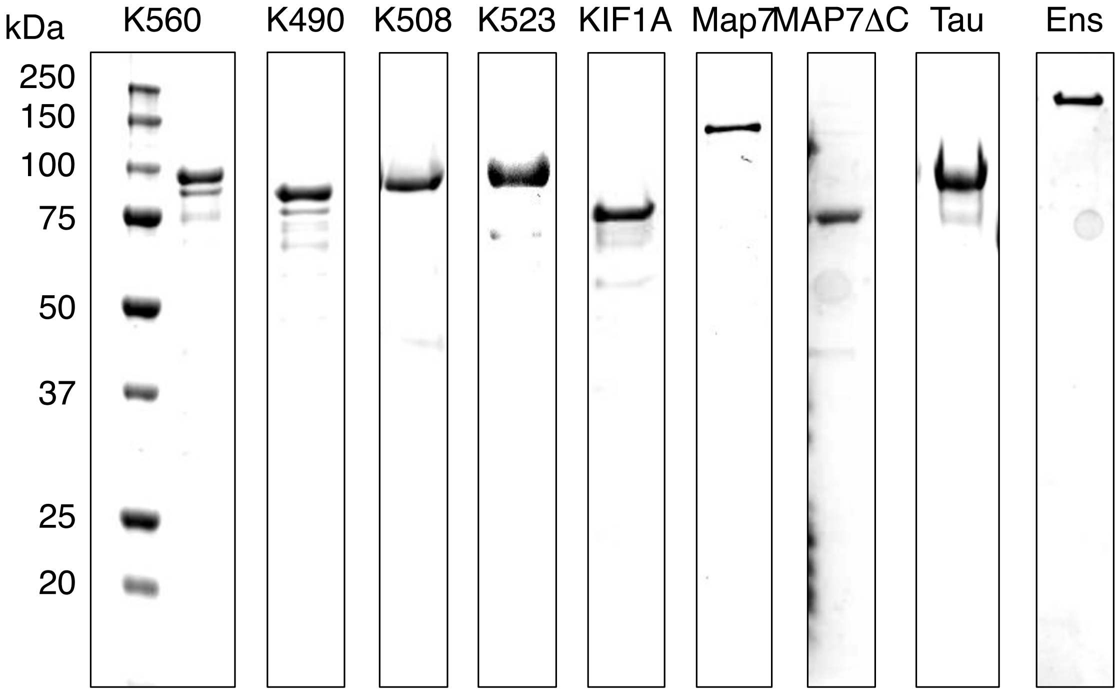

Supplementary Figure 1. Purified recombinant proteins used in this study.

Coomassie Blue-stained SDS-PAGE gels of K560-mScarlet, K490-mScarlet, K508-mScarlet, K523-mScarlet, KIF1A-mScarlet, sfGFP-MAP7, sfGFP-MAP7ΔC, mTagBFP-tau, and sfGFP-ensconsin.

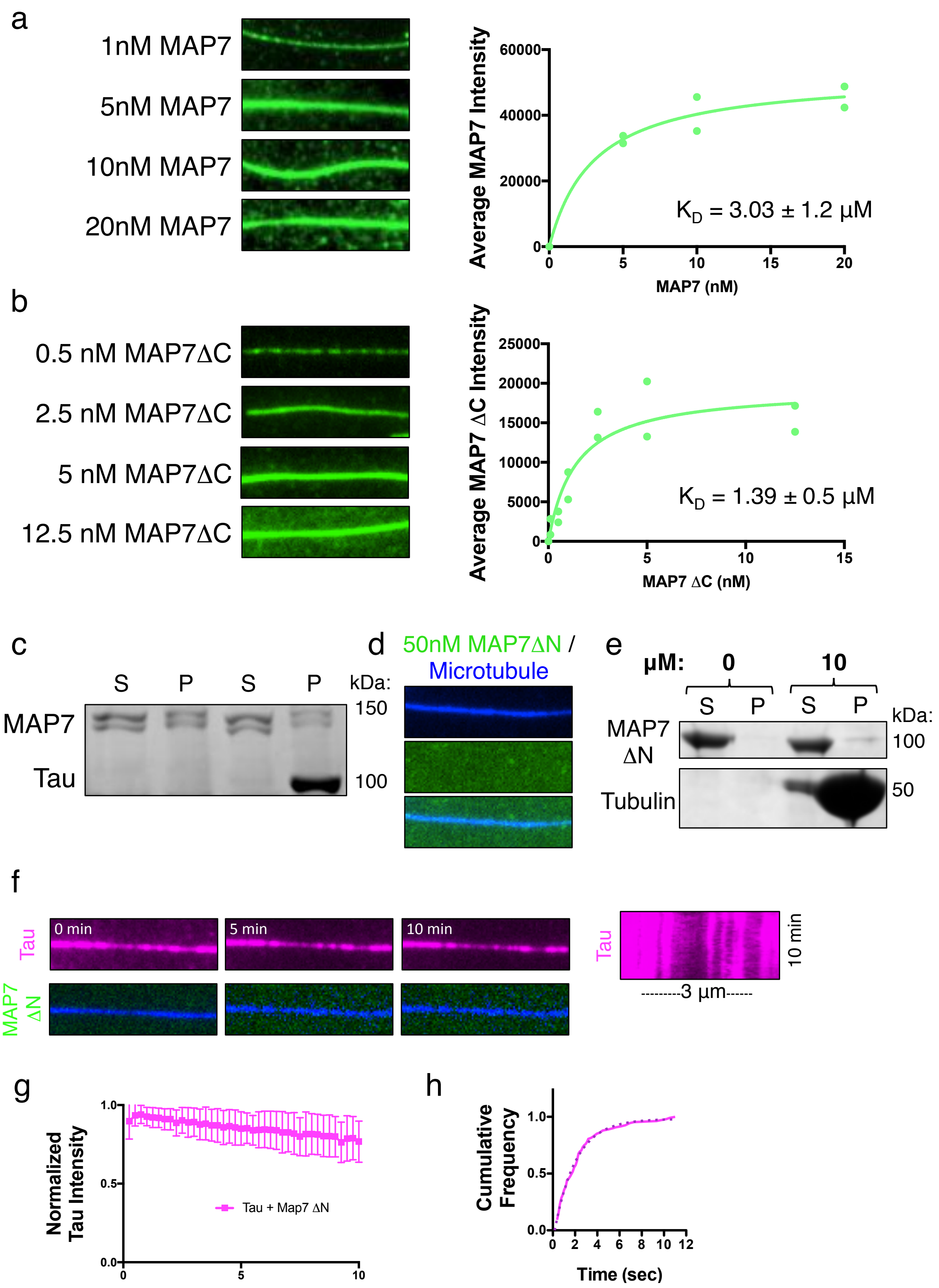

Supplementary Figure 2. Analysis of MAP7 and tau competition on the microtubule lattice.

(a-b) Fluorescence images of either full-length sfGFP-MAP7 (**a**) or sfGFP-MAP7ΔC (**b**) bound to microtubules, and corresponding quantification of fluorescence intensity of microtubule-bound sfGFP-MAP7 or sfGFP-MAP7ΔC plotted against concentration (full-length MAP7: K_D ± s.d. = 3.03 ± 1.2; n = 48, 21, 48, 44, and 49 microtubules for 0 nM, 1 nM, 5 nM, 10 nM, and 20 nM concentrations, respectively from three independent trials; MAP7ΔC K_D ± s.d. = 1.39 ± 0.5; n = 24 microtubules for each concentration 0 nM, 0.1 nM, 0.5 nM, 1 nM, 2.5 nM, 5 nM, 12.5 nM, from two independent trials; $P = 0.177$). **(c)** Coomassie-Blue stained SDS-PAGE gel of GFP binding protein (GBP) pull-down with purified recombinant proteins. 500 nM sfGFP-tau was used for pull-down assays with 250 nM TagRFP-MAP7, (n = three independent trials). S = supernatant and P = pellet. **(d)** Fluorescence images of sfGFP-MAP7ΔN not bound to the microtubule. Images are 3.1 μm. **(e)** Coomassie-Blue stained SDS-PAGE gel of 500 nM MAP7ΔN in the absence or presence of taxol-stabilized microtubules. S = supernatant and P = pellet. **(f)** Successive movie frames of mScarlet-tau in the presence of sfGFP-MAP7ΔN. Images are 3.1 μm. The corresponding kymograph to the right depicts tau over 10 min (images were acquired at 15 s intervals). **(g)** Quantification of tau fluorescence intensity in the presence of MAP7ΔN over 10 min (n = 14 microtubules from two independent trials). **(h)** Adjusted scale graph depicting the cumulative frequency of tau (decay constant, $\tau = 1.9 \pm 0.1$ s; n = 142 events from n = 16 microtubules from two independent trials) dwell times fit to a one phase exponential decay ($R^2 = 0.995$ for tau $P < 0.0001$).

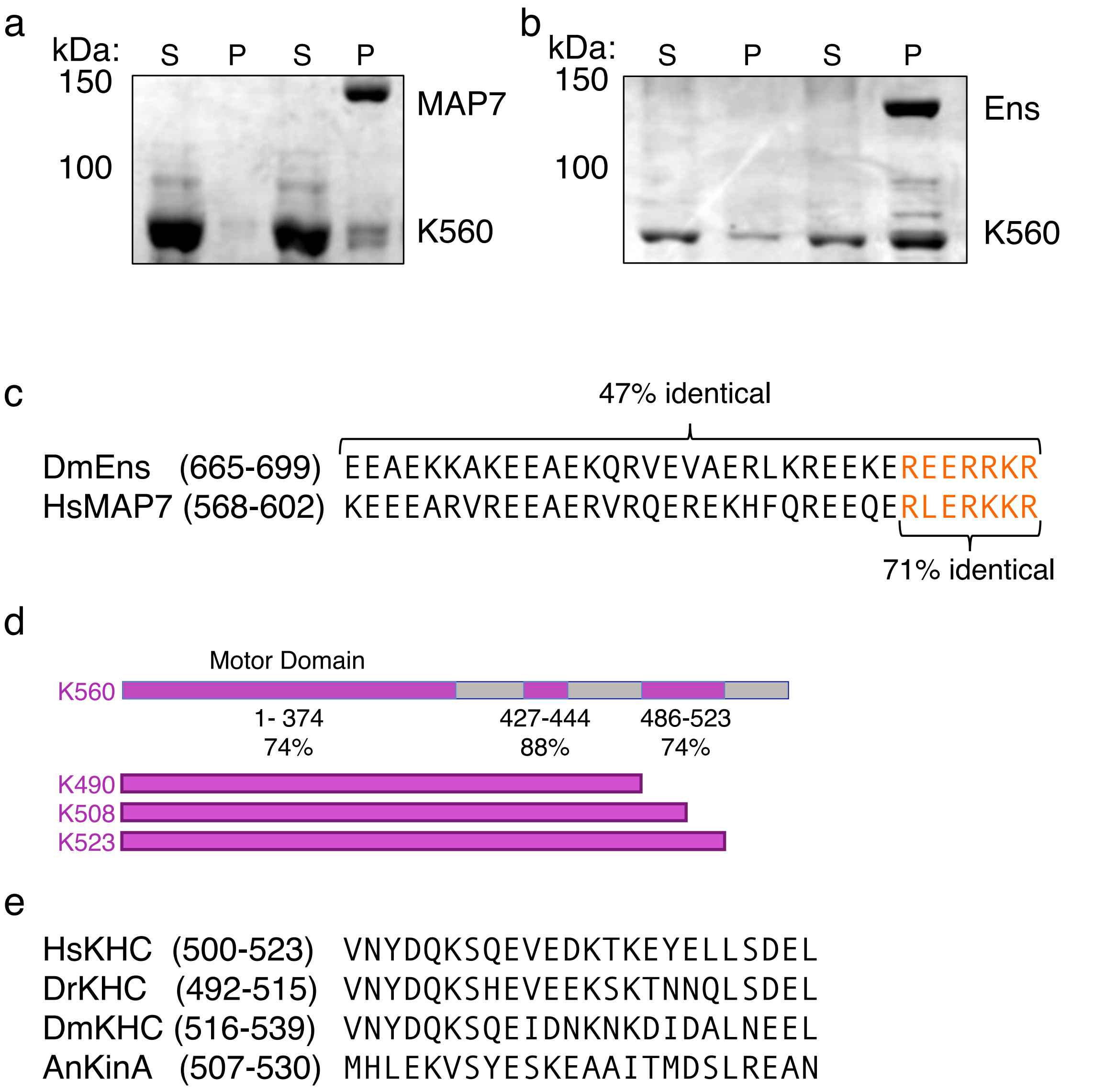

Supplementary Figure 3. Dissection of the MAP7/ensconsin interaction with kinesin-1.

(a-b) Coomassie Blue-stained SDS-PAGE gels of FLAG pull-downs with purified recombinant proteins. Either full-length sfGFP-MAP7-FLAG **(c)** or sfGFP-Ensconsin-FLAG were used for pull-down assays with K560-mScarlet (n = two independent trials per assay). S = supernatant and P = pellet. **(c)** Sequence alignment between *Drosophila melanogaster* ensconsin (DmEns) and *Homo sapiens* MAP7 (HsMAP7) with the percent

identity indicated. **(d)** Schematic diagram of K560 and the associated constructs used in the mapping studies of Fig. 4i-j. The percent identities indicate the conservation between DmEns and HsMAP7. **(e)** Sequence alignment between HsKHC, *Danio rerio* KHC (DrKHC), DmKHC, and *Aspergillus nidulans* KinA (AnKinA).

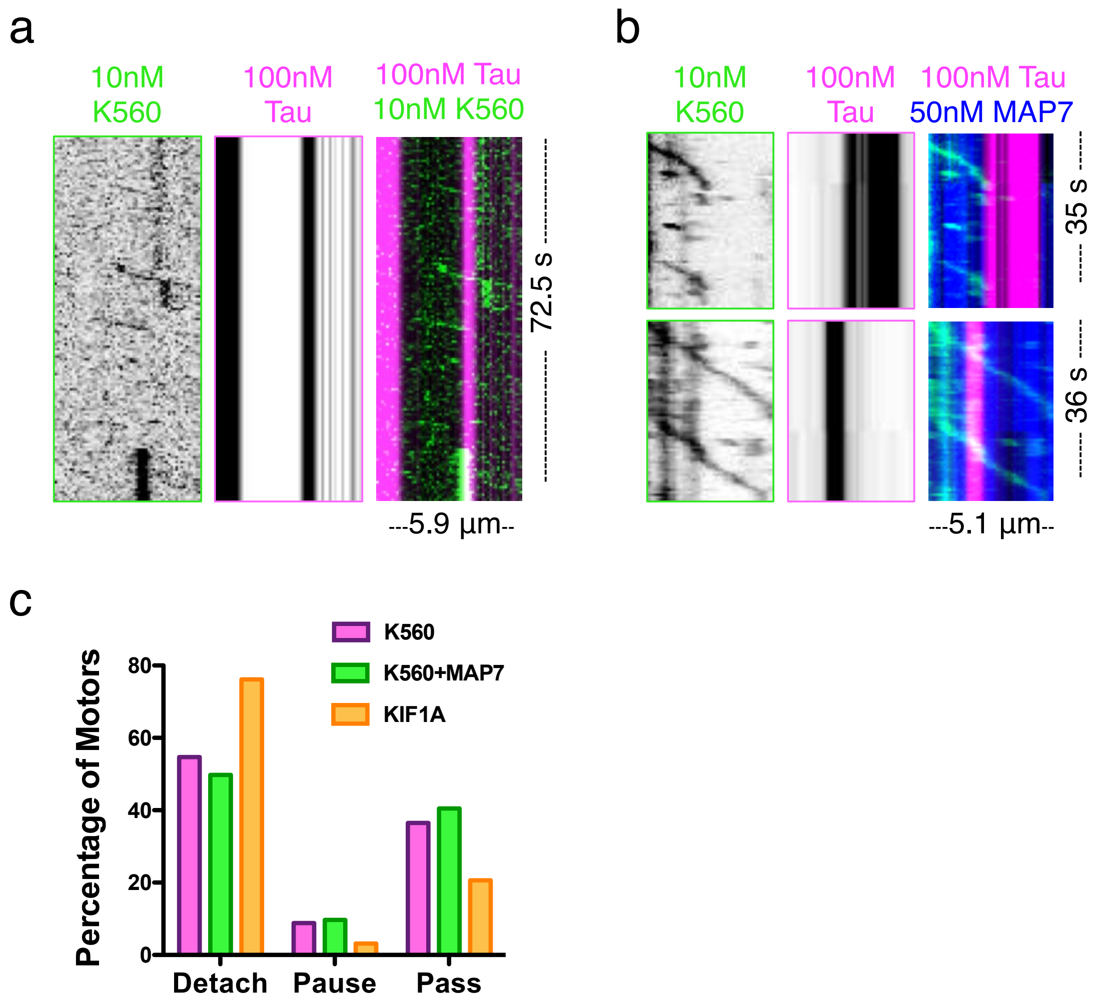

Supplementary Figure 4. The effects of tau on kinesin-1 in the absence and presence of MAP7.

(a-b) Kymographs depicting the effect of 100 nM mTagBFP-tau (pink) on 10 nM K560-mScarlet (green) motility in the absence **(a)** or presence **(b)** of 50 nM sfGFP-MAP7 (blue). **(c)** Quantification of the percent of K560 motors that detach, pause, or pass tau patches in the absence (pink) or presence (green) of MAP7 (54.7 % vs. 49.8 % detach, 8.8 % vs. 9.7 % pause, and 36.5 % vs. 40.5 % pass for K560 alone vs. K560 + MAP7, respectively; n = 159 from two independent experiments and 227 events from three independent experiments for K560 alone vs. K560 + MAP7, respectively). Related to Fig.

5d, also shown is the quantification of percent of KIF1A motors (orange) that detach, pause, or pass tau patches (76.2 % detach, 3.2 % pause, and 20.6 % pass; n = 126 motors from two independent experiments).

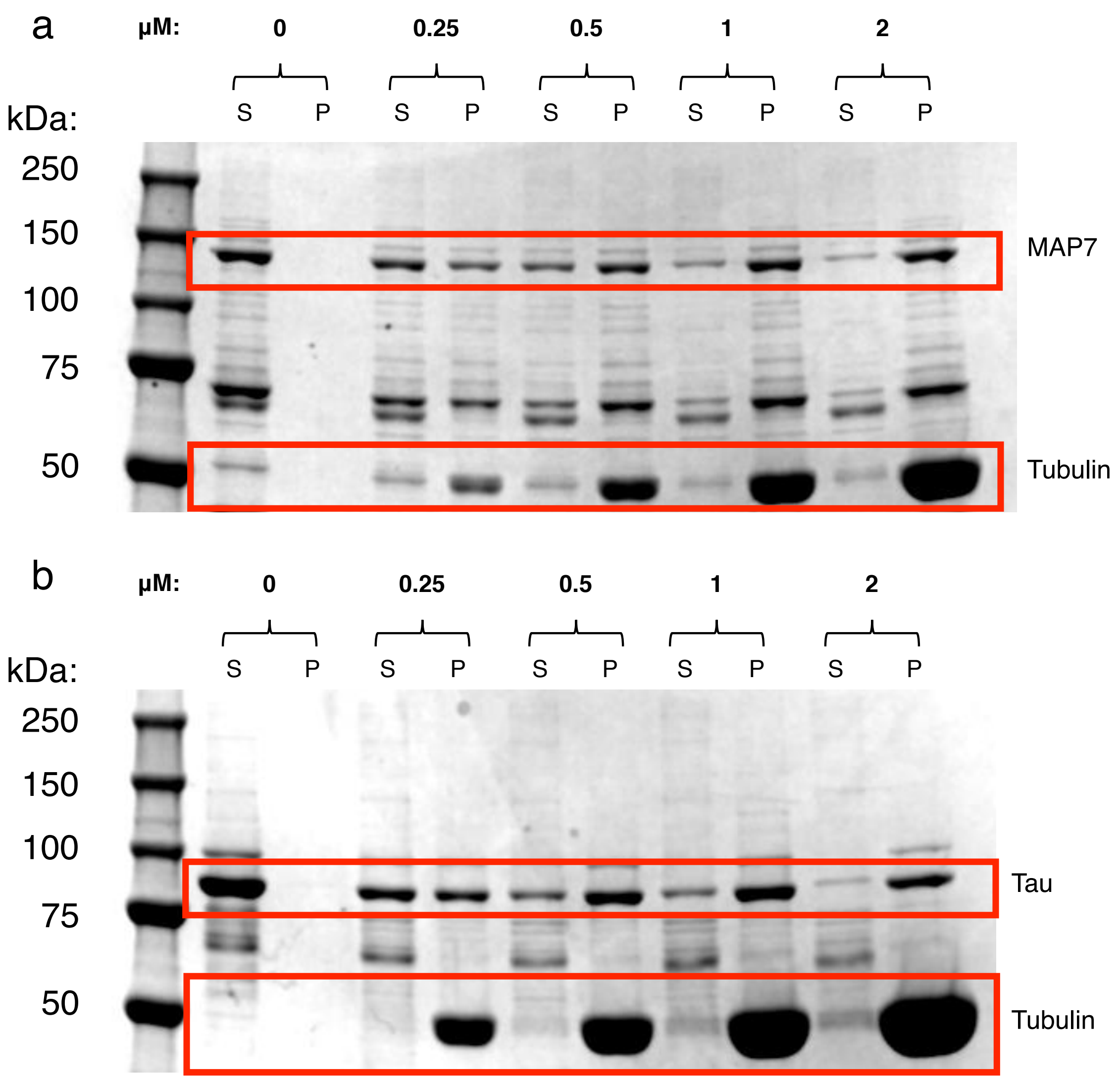

Supplementary Figure 5. Uncropped images of SDS-PAGE gels shown in Figure 2i for MAP7 (a) and tau (b) microtubule co-pelleting assays. Red boxes highlight the portions of the gels that are shown in the figure. Ladder is shown on the left.
